# tRNA modification enzyme MiaB connects environmental cues to activation of *Pseudomonas aeruginosa* type III secretion system

**DOI:** 10.1371/journal.ppat.1011027

**Published:** 2022-12-05

**Authors:** Qiqi Lin, Jiahui Huang, Zhiqing Liu, Qunyi Chen, Xinbo Wang, Guohui Yu, Ping Cheng, Lian-Hui Zhang, Zeling Xu

**Affiliations:** 1 Guangdong Province Key Laboratory of Microbial Signals and Disease Control, Integrative Microbiology Research Centre, South China Agricultural University, Guangzhou, China; 2 Guangdong Laboratory for Lingnan Modern Agriculture, South China Agricultural University, Guangzhou, China; 3 School of Food Pharmaceutical Engineering, Zhao Qing University, Zhaoqing, China; 4 Institute of Plant Health, Zhongkai University of Agriculture and Engineering, Guangzhou, China; University of Toronto, CANADA

## Abstract

*Pseudomonas aeruginosa*, a major inhabitant of numerous environmental reservoirs, is a momentous opportunistic human pathogen associated with severe infections even death in the patients suffering from immune deficiencies or metabolic diseases. Type III secretion system (T3SS) employed by *P*. *aeruginosa* to inject effector proteins into host cells is one of the pivotal virulence factors pertaining to acute infections caused by this pathogen. Previous studies showed that *P*. *aeruginosa* T3SS is regulated by various environmental cues such as calcium concentration and the host signal spermidine. However, how T3SS is regulated and expressed particularly under the ever-changing environmental conditions remains largely elusive. In this study, we reported that a tRNA modification enzyme PA3980, designated as MiaB, positively regulated T3SS gene expression in *P*. *aeruginosa* and was essential for the induced cytotoxicity of human lung epithelial cells. Further genetic assays revealed that MiaB promoted T3SS gene expression by repressing the LadS-Gac/Rsm signaling pathway and through the T3SS master regulator ExsA. Interestingly, *ladS*, *gacA*, *rsmY* and *rsmZ* in the LadS-Gac/Rsm signaling pathway seemed potential targets under the independent regulation of MiaB. Moreover, expression of MiaB was found to be induced by the cAMP-dependent global regulator Vfr as well as the spermidine transporter-dependent signaling pathway and thereafter functioned to mediate their regulation on the T3SS gene expression. Together, these results revealed a novel regulatory mechanism for MiaB, with which it integrates different environmental cues to modulate T3SS gene expression in this important bacterial pathogen.

## Author summary

T3SS in *P*. *aeruginosa* is a predominant virulence factor causing acute infections in many hosts including human beings. Expression of T3SS genes is usually controlled by an intrinsic ExsECDA partner-switching cascade together with numerous extrinsic regulators with most of them are related to or inducible by different environmental cues. Whether any intermediate players exist to connect environmental cues to the activation of *P*. *aeruginosa* T3SS is not known. Here we demonstrated that a tRNA modification enzyme MiaB was upregulated by Vfr and the spermidine transporter-dependent signaling pathway, two important T3SS regulators in response to various host-related environments, to mediate their regulation on T3SS gene expression through the LadS-Gac/Rsm signaling pathway and the T3SS master regulator ExsA. Findings in this study not only advanced our understanding on the complex T3SS gene regulation in response to different environmental cues but also provided important targets for future anti-infective exploitations.

## Introduction

*Pseudomonas aeruginosa* is a highly adaptive and ubiquitous Gram-negative opportunistic pathogen, leading to various acute and chronic infections in immune-compromised individuals such as those with cystic fibrosis, burn wound and cancer diseases [1,2]. *P*. *aeruginosa* encompasses numerous virulence factors, including flagella, pili, type III secretion system (T3SS), exotoxin, mucoid exopolysaccharide alginate and so forth, which collectively contribute to its pathogenicity [3,4]. Among them, T3SS is one of the key virulence determinants playing a critical role in the bacterial acute infection and protection of the pathogen from phagocytosis in the process of host-pathogen interaction [5–7]. *P*. *aeruginosa* is one of the few notorious pathogens causing severe nosocomial infections [8], and infections caused by *P*. *aeruginosa* are hard to eliminate owing to its strong environmental adaptability and extensive drug resistance against multiple antibiotics [9]. These highlight the importance and urgent need to elucidate the molecular mechanisms that how host-related environments reprogram the expression of virulence factor genes and promote the bacterial virulence in *P*. *aeruginosa*.

T3SS is a widely conserved molecular syringe in gram-negative bacteria that injects effector proteins directly into cytosols of host cells, which facilitates bacterial infection by interfering the normal physiological process and immune responses of host organisms [10]. Up to present, four effector proteins (ExoS, ExoT, ExoU, ExoY) have been identified and characterized in *P*. *aeruginosa* [11,12]. T3SS gene expression is concurrently regulated by a range of intrinsic and extrinsic regulators [13]. The transcriptional regulator ExsA, encoded by the last gene of the operon *exsCEBA*, is recognized as the master regulator of the entire T3SS regulatory network and can self-regulate its own operon *exsCEBA* [14]. ExsA-based intrinsic regulation of T3SS is achieved by a group of regulatory proteins consisting of ExsA, ExsC, ExsD and ExsE with a partner-switching mechanism [15]. In detail, under non-inducible conditions such as high calcium concentration or absence of potential host cell contact, the negative regulatory protein ExsE is accumulated and binds to the positive regulatory protein ExsC in the cytoplasm at a 1:2 ratio, which releases ExsD to sequester ExsA by forming a heterodimer with it, keeping the T3SS gene expression in a repressed state and the T3SS channel in a closed state. On the contrary, under T3SS inducible conditions, secretion of ExsE lowers the intracellular ExsE level, which triggers the formation of ExsD/ExsC complex and release of ExsA to activate the expression of T3SS genes [16–19]. An important feature of this regulatory cascade is that small changes in ExsC, ExsE, ExsA or ExsD levels can have a profound effect on T3SS gene expression [16,20], which endows *P*. *aeruginosa* the ability to respond to the changing environmental conditions in a quick and efficient manner and thus switch on or off T3SS gene expression accordingly.

A range of environmental cues have been reported to affect the expression of T3SS genes in *P*. *aeruginosa* such as host cell contact, serum and calcium depletion, osmotic stress, oxygen depletion [21–25]. Extensive efforts to realize the signaling systems that transduce environmental signals to T3SS gene expression have been paid and remarkable progress has been achieved so far. For example, Vfr is appreciated as an important global regulator of virulence factor genes including T3SS, which was reported to regulate T3SS gene expression by directly activating the transcription of *exsA* [26]. The activity of Vfr relies on the intracellular level of the second messenger cyclic AMP (cAMP) and the production of intracellular cAMP is affected by various host-related environmental conditions such as osmotic stress, calcium depletion and another second messenger cyclic di-GMP (c-di-GMP) [27, 28]. In addition to Vfr, the RetS/LadS/Gac/Rsm system is known as another critical regulatory system involved in modulating *exsA* transcription [29–32]. RsmA is a CsrA family protein that binds target mRNAs through the recognition of CGA motifs in the loop of stem-loop structures, stimulating the expression of virulence factors related to acute infections such as T3SS [33,34]. The activity of RsmA is antagonized by two small RNAs, *rsmY* and *rsmZ*, whose expression is regulated by the two-component system GasS/GacA [35]. Two additional histidine kinases, LadS and RetS, are known to inversely regulate the Gac/Rsm signaling cascade by interfering with the GacS activity [36,37]. LadS is a calcium-responsive histidine kinase that plays an important role in the transition of acute-chronic behavior during infections by monitoring surrounding calcium concentration [30]. Moreover, many other factors controlling the *exsA* transcription and translation in *P*. *aeruginosa* have been identified such as the nucleoid-associated protein Fis [38], the AraC family transcriptional regulator VqsM [39], the H-NS family members MvaT and MvaU [40], the RNA helicase DeaD [41].

Previously, we demonstrated that spermidine produced by host cells acts as a cross-kingdom communication signal to activate T3SS gene expression in *P*. *aeruginosa* through a spermidine-specific ABC transporter SpuDEFGH [42], and follow-up development of antibodies to immunologically block the spermidine-mediated host-pathogen interaction has been proved as an effective strategy to prevent and control *P*. *aeruginosa* infections [43,44]. These investigations not only expanded our understanding of diverse environmental cues that influence T3SS gene expression in *P*. *aeruginosa* but also highlighted their importance for anti-infective applications. Nonetheless, whether additional factors connect environmental cues to T3SS gene expression is scarcely known and deserves more explorations. In this study, we identified a tRNA modification enzyme PA3980 (MiaB) that mediates isopentenyladenosine (i^6^A) modification is a novel regulator for T3SS gene expression in *P*. *aeruginosa*. i^6^A modification has been shown previously to be associated with the regulation of morphogenesis and secondary metabolism in *Streptomyces* [45], iron accumulation related pathogenicity in *E*. *coli* [46], production of virulence factors in *Shigella flexneri* [47], and rapamycin sensitivity in *Schizosaccharomyces pombe* through altering the paring accuracy of amino acid sequences [48]. Here, we further reported that MiaB is required for mediating the regulation of cAMP-dependent regulator Vfr and the spermidine transporter-dependent signaling pathway on T3SS gene expression. Findings from this study unveiled a new player connecting various environmental cues to T3SS gene expression in *P*. *aeruginosa* and provided a new insight into the complicated and sophisticated regulatory mechanisms that govern T3SS activation in *P*. *aeruginosa*.

## Results

### Identification of a novel positive regulator PA3980 (MiaB) of *exsCEBA* transcription in *P*. *aeruginosa*

**Fig 1:**
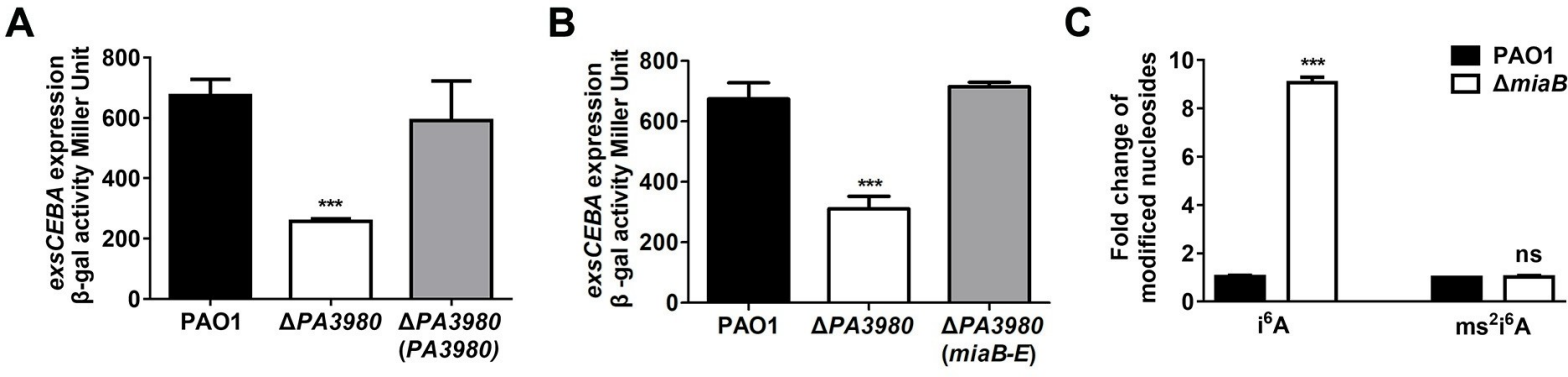
The T3SS operon *exsCEBA* in *P*. *aeruginosa* was positively regulated by an adenosine tRNA methylthiotransferase PA3980 (MiaB). (**A**) *β*-galactosidase activity of the P*exsCEBA*-*lacZ* transcriptional fusion in the wide-type PAO1 strain, Δ*PA3980*, and Δ*PA3980* with *in trans* expression of *PA3980*. (**B**) *β*-galactosidase activity of the P*exsCEBA*-*lacZ* transcriptional fusion in the wide-type PAO1 strain, Δ*PA3980*, and Δ*PA3980* with *in trans* expression of *miaB* from *E*. *coli* (*miaB-E*). (**C**) LC-MS measurement of the tRNA A37 *N*^6^-isopentenyladenosine (i^6^A) and 2-methylthio-*N*^6^-isopentenyladenosine (ms^2^i^6^A) in the wide-type PAO1 strain and Δ*PA3980* mutant. ns, not significant; ***, *P* < 0.001 compared to the wild-type PAO1 strain based on Student’s *t* test.

Our previous transposon mutagenesis screening assay using a P*exsCEBA*-*lacZ* reporter strain PAO1pClacZ (pCZ) discovered that a mutant with transposon insertion at the gene locus PA3980 displayed reduced activity of the *exsCEBA* promoter [42], suggesting a potentially new regulatory route for T3SS gene activation in *P*. *aeruginosa*. To first verify the impact of PA3980 on *exsCEBA* expression, we generated an in-frame deletion mutant Δ*PA3980* from the parental strain pCZ and measured the expression of *exsCEBA* by *β*-galactosidase activity assay in the LBN (LB supplemented with 7.5 mM NTA) medium which was conducive for T3SS activation [42]. As shown in Fig 1A, transcription activity of the *exsCEBA* promoter in the Δ*PA3980* mutant was significantly reduced compared to the parental wild-type PAO1 strain. *in trans* expression of *PA3980* in the Δ*PA3980* mutant fully restored the transcriptional level of *exsCEBA* to the wild-type strain level (Fig 1A), confirming that PA3980 exhibited a positive impact on the transcription of the *exsCEBA* operon.

PA3980 is a hypothetic protein which has not yet been characterized previously. Alignment of the amino acid sequences showed that PA3980 in *P*. *aeruginosa* shared 68.03% and 68.48% identity with the MiaB proteins from *E*. *coli* and *S*. typhimurium, respectively (S1 Fig). In these two bacterial pathogens, MiaB was known as a radical *S*-adenosylmethionine (SAM) methylthiotransferase to catalyze the final step in the biosynthesis of the hypermodified nucleoside 2-methylthio-*N*^6^-isopentenyladenosine (ms^2^i^6^A) from i^6^A [49]. Given that the high identity between PA3980 and MiaB in other pathogens, we next constructed a plasmid (pBBR1-*miaB*-*E*) to carry the *miaB* gene from *E*. *coli* and transformed the plasmid into the Δ*PA3980* mutant to see whether expression of an exogenous MiaB could compensate for the loss-of-function of PA3980. As we expected, ectopic expression of the *E*. *coli miaB* in Δ*PA3980* completely recovered the transcription level of *exsCEBA* back to the level in the wild-type strain (Fig 1B), which indicated that the positive regulator PA3980 of *exsCEBA* transcription could be a tRNA modification enzyme. To further characterize the biological function of PA3980, total RNA of the wild-type PAO1 and Δ*PA3980* strains were extracted for tRNA nucleotide modification analysis. LC-MS measurement showed that the substrate nucleoside i^6^A was accumulated about 9 folds when *PA3980* was deleted (Fig 1C), demonstrating that PA3980 functioned to catalyze i^6^A modification as MiaB in other pathogens. However, the product of nucleoside ms^2^i^6^A in Δ*PA3980* did not decrease significantly compared with the wild-type PAO1 strain (Fig 1C), implying the presence of additional tRNA modification enzymes in *P*. *aeruginosa* to conduct the formation of ms^2^i^6^A from unknown pathways. Together, the *exsCEBA* activator PA3980 in PAO1 was functionally characterized as an adenosine tRNA methylthiotransferase and designated as MiaB hereafter.

tRNA modifications frequently play pleiotropic roles in regulating bacterial physiology [45,50]. Owing that MiaB was essential for i^6^A modification, we speculated that its activation on *exsCEBA* transcription could be associated with the level of modified tRNAs. To test this speculation, we deleted another gene *miaA* which encodes tRNA isopentenyltransferase for the generation of i^6^A and examined whether the transcription of *exsCEBA* was influenced by the deletion of *miaA*. Different from *miaB*, the activity of *exsCEBA* transcription was unchanged with the deletion of *miaA* despite the Δ*miaA* mutant showed significantly decreased intracellular content of i^6^A compared to the wild-type PAO1 strain (S2 Fig). These results indicated that MiaB regulated *exsCEBA* transcription through a new pathway that was independent of tRNA modifications.

### MiaB was required for T3SS gene expression and the full virulence of *P*. *aeruginosa*

**Fig 2:**
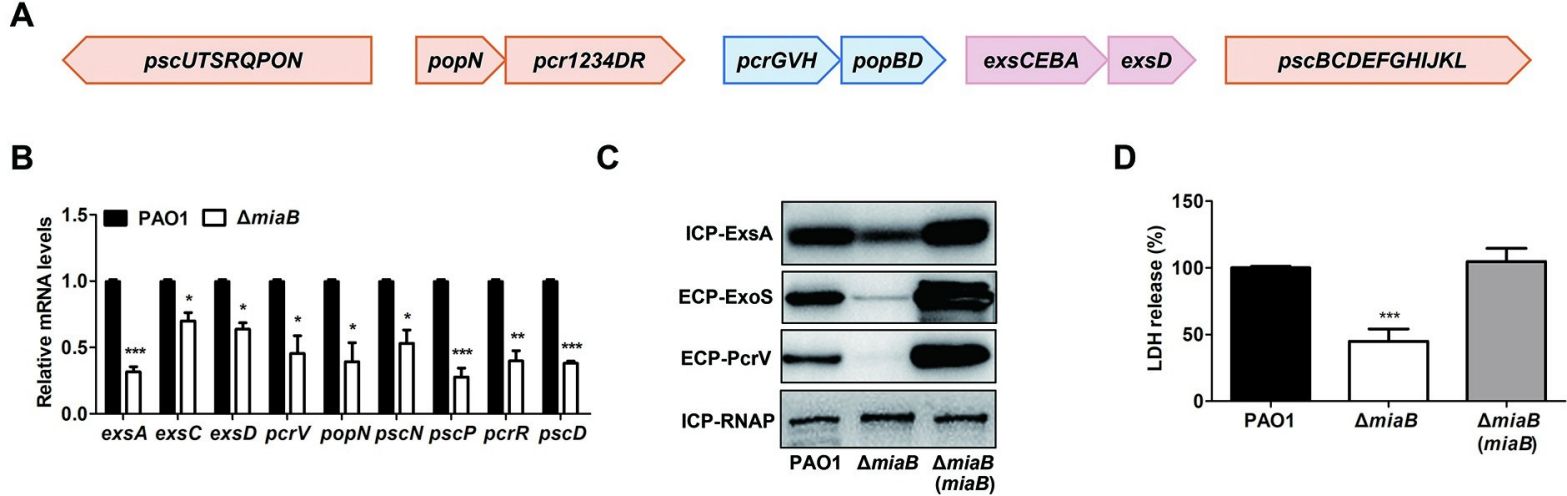
MiaB positively regulated T3SS gene expression and was necessary for the virulence in *P*. *aeruginosa*. (**A**) Five clustered operons involved in the T3SS in *P*. *aeruginosa*. Orange: needle complex; Blue: translocation apparatus; Pink: regulation. (**B**) Relative expression of T3SS genes in the wild-type PAO1 and Δ*miaB* mutant measured by RT-qPCR. (**C**) Productions of the cytosol ExsA protein and the secreted ExoS and PcrV proteins determined by western blot in the strains of wide-type PAO1, Δ*miaB*, and Δ*miaB* with *in trans* expression of *miaB*. ECP: extracellular protein, ICP: intracellular protein. RNA polymerase (RNAP) was used as an internal control for ICP. ECPs were normalized with the cell density. (**D**) Cytotoxicity was evaluated by monitoring LDH release from the A549 cells infected by the strains of wide-type PAO1, Δ*miaB*, and Δ*miaB* with *in trans* expression of *miaB*. *, *P* < 0.05; **, *P* < 0.01; ***, *P* < 0.001 compared to the wild-type PAO1 strain based on Student’s *t* test.

To further examine the role of MiaB in regulating T3SS gene expression, we determined expression of more T3SS-related genes that encode regulatory proteins (*exsA*, *exsC*, *exsD*), translocation apparatus proteins (*pcrV*), and needle complex proteins (*pcrR*, *popN*, *pscN*, *pscP*, *pscD*) in the wild-type PAO1 and the Δ*miaB* strains after they were cultured in the LBN medium (Fig 2A). RT-qPCR analysis showed that the RNA levels of all the tested T3SS genes such as *exsA*, *exsC*, *exsD*, *pcrV*, *pcrR*, *popN*, *pscN*, *pscP* and *pscD* were remarkably lower in the Δ*miaB* mutant than that in the wild-type PAO1 strain (Fig 2B). To further confirm the regulation at translational level, representative cytosol and secreted T3SS proteins such as ExsA, ExoS and PcrV were selected and examined using the western blot assay. It was shown that deletion of *miaB* led to an obvious decrease of the cytosol ExsA at the protein level (Fig 2C). Similarly, substantial reduction of the secreted proteins ExoS and PcrV were observed in the mutant Δ*miaB* (Fig 2C). Amounts of all three proteins in the Δ*miaB* mutant could be restored by the *in trans* expression of *miaB* (Fig 2C). These results further demonstrated that MiaB was a positive regulator of the T3SS gene expression in *P*. *aeruginosa*. Owing that T3SS plays a crucial role in pathogenesis during host infections [23], we surmised that the attenuated T3SS gene expression caused by deletion of MiaB would result in defective cytotoxicity of the pathogen. To test this, we infected human lung epithelial cell A549 with the wild-type PAO1 strain, Δ*miaB* mutant and the mutant with *in trans* expression of *miaB* (Δ*miaB*(*miaB*)). Consistent with the expression levels of T3SS genes in these strains, Δ*miaB* displayed significantly lower cytotoxicity than the wild-type strain and *in trans* expression of *miaB* in the mutant fully restored the cell cytotoxicity (Fig 2D). These results substantiated that loss of *miaB* in *P*. *aeruginosa* exerted a serious impact on the T3SS gene expression as well as the T3SS-associated virulence and pathogenicity of this pathogen.

### MiaB modulated T3SS gene expression in the ExsA-dependent manner

**Fig 3:**
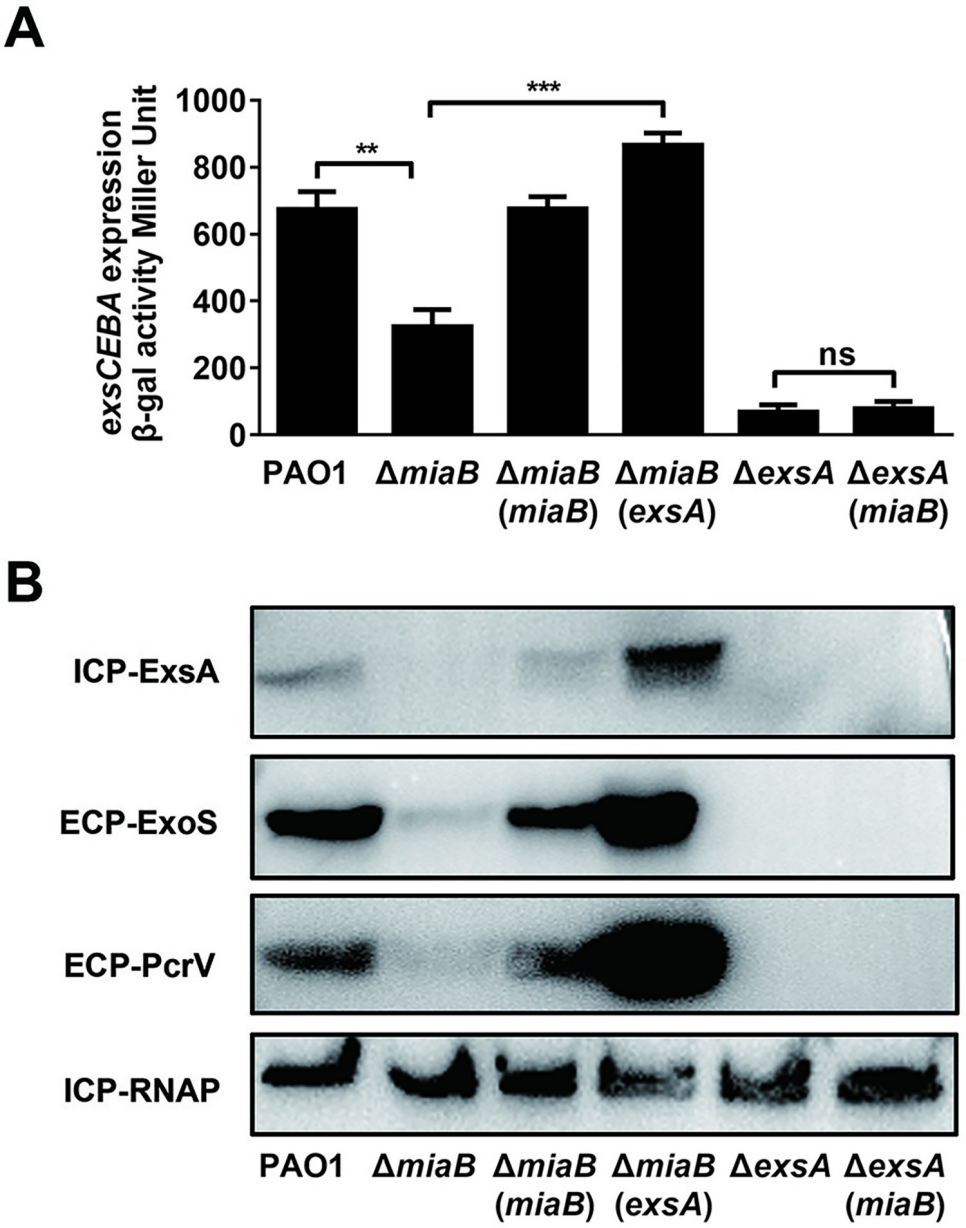
MiaB modulated T3SS gene expression through the master regulator ExsA. (**A**) *β*-galactosidase activity of the P*exsCEBA*-*lacZ* transcriptional fusion in the wide-type PAO1 strain, Δ*miaB*, Δ*exsA* mutants and mutants with *in trans* expression of *exsA* or *miaB*. (**B**) Productions of ExsA, ExoS and PcrV proteins measured by western blot in the wide-type PAO1 strain, Δ*miaB*, Δ*exsA* mutants and mutants with *in trans* expression of *exsA* or *miaB*. ns, not significant; **, *P* < 0.01; ***, *P* < 0.001 compared to the indicated group based on Student’s *t* test.

We next attempted to further investigate the regulatory circuit between MiaB and T3SS gene expression. Since ExsA is a pivotal regulator of T3SS in *P*. *aeruginosa* and its transcription level was reduced in the absence of *miaB* (Fig 2B), we then asked whether MiaB was a novel extrinsic regulator promoting T3SS gene expression through the intrinsic regulator ExsA. It was shown that *in trans* expression of *exsA* in Δ*miaB* rescued the repression of the promoter activity of the *exsCEBA* operon and protein productions of ExsA, ExoS and PcrV (Fig 3A and 3B). In contrast, *in trans* expression of *miaB* in Δ*exsA* did not change the promoter activity of *exsCEBA* and the translation of T3SS-associated proteins in this mutant (Fig 3A and 3B). These results indicated that MiaB was an extrinsic regulator that modulated T3SS gene expression through the T3SS master regulator ExsA. Transcription of *exsA* was driven from two distinct promoters. In addition to the promoter of *exsCEBA* which produces the *exsCEBA* polycistronic mRNA, promoter of *exsA* also generates a monocistronic *exsA* mRNA [26]. So, we further measured the promoter activity of *exsA* to determine if MiaB induced the transcription of *exsA* as well by directly activating its own promoter. *β*-galactosidase activity assay did not show any difference on the *exsA* promoter activity between the wild-type and Δ*miaB* strains (S3 Fig), demonstrating that MiaB promoted *exsA* transcription by only inducing the *exsCEBA* operon.

### MiaB regulated T3SS gene expression by recruiting the histidine kinase LadS

**Fig 4:**
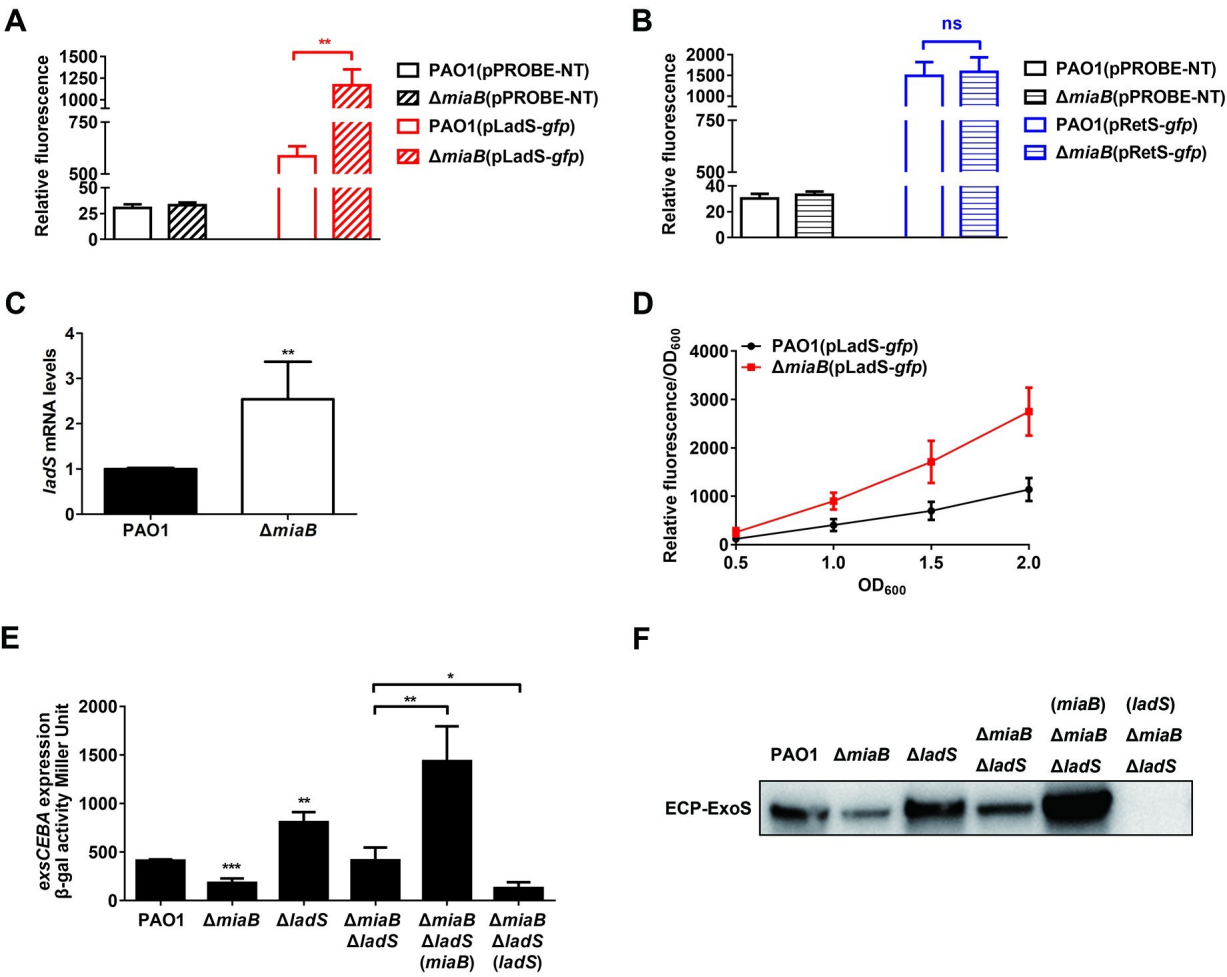
LadS was involved in the MiaB-mediated regulation of T3SS gene expression. (**A**) Relative fluorescence intensity of the pLadS-*gfp* transcriptional fusion measured in the wild-type PAO1 strain and the Δ*miaB* mutant. (**B**) Relative fluorescence intensity of the pRetS-*gfp* transcriptional fusion measured in the wild-type PAO1 strain and the Δ*miaB* mutant. (**C**) Relative expression of the *ladS* gene in the wild-type PAO1 and Δ*miaB* mutant measured by RT-qPCR. (**D**) Relative fluorescence intensity of the pLadS-*gfp* transcriptional fusion measured in the wild-type PAO1 strain and the Δ*miaB* mutant when cell growth at the OD_600_ of 0.5, 1.0, 1.5 and 2.0. (**E**) *β*-galactosidase activity of the P*exsCEBA*-*lacZ* transcriptional fusion in the wide-type PAO1 strain, Δ*miaB*, Δ*ladS* and the Δ*miaB*Δ*ladS* mutants as well as the Δ*miaB*Δ*ladS* mutant with *in trans* expression of *miaB* and *ladS*. (**F**) Production of the ExoS protein measured by western blot in the wide-type PAO1 strain, Δ*miaB*, Δ*ladS* and the Δ*miaB*Δ*ladS* mutants as well as the Δ*miaB*Δ*ladS* mutant with *in trans* expression of *miaB* and *ladS*. ns, not significant; *, *P* < 0.05; **, *P* < 0.01; ***, *P* < 0.001; compared to the wild-type PAO1 strain or indicated group based on Student’s *t* test.

Because the RetS/LadS/Gac/Rsm system is one of the critical signaling system influencing T3SS gene expression in *P*. *aeruginosa* and the hybrid sensor histidine kinases RetS and LadS are located at the top of this signaling cascade [51,52], we moved to determine the relationship between MiaB and RetS or LadS in the modulation of T3SS gene expression. We first amplified the promoter regions of *retS* and *ladS* to generate the promoter-*gfp* fusions pRetS-*gfp*, and pLadS-*gfp*, respectively, based on a transcription fusion vector pPROBE-NT. Next, these promoter activity reporters including the vector control were transformed into the wild-type PAO1 strain and the Δ*miaB* mutant. Activities of the transcription fusions were measured using a CytoFLEX flow cytometer after the cells were collected at the exponential phase with OD_600_ of 1.2–1.5. The results showed that the activity of the *ladS* promoter in Δ*miaB* was enhanced significantly compared to that in wild-type PAO1, whereas no obvious change of the *retS* promoter was noticed between both strains (Fig 4A and 4B). Increased RNA level of *ladS* confirmed its upregulation in the absence of MiaB (Fig 4C). Time course analysis showed that the activity of the *ladS* promoter was continually enhanced in Δ*miaB* since OD_600_ was 0.5 without any growth difference compared to the wild-type PAO1 strain (Figs 4D and S4). To determine whether reduced T3SS gene expression in Δ*miaB* was due to the elevated LadS level, we deleted *lasS* in the background of Δ*miaB*. Activity of the *exsCEBA* promoter and a representative protein level of ExoS were next measured, which showed that further deletion of *ladS* restored the *exsCEBA* promoter activity and ExoS production in Δ*miaB* back to the wild-type level (Fig 4E and 4F). These results together suggested that MiaB modulated T3SS gene expression by recruiting the LadS. However, from another aspect, further deletion of *miaB* in the Δ*ladS* mutant also led to a decrease of the *exsCEBA* promoter activity and the ExoS production compared to the *ladS* single deletion mutant Δ*ladS* (Fig 4E and 4F). Moreover, *in trans* expression of *miaB* in the Δ*miaB*Δ*ladS* strain still led to the induction of the *exsCEBA* promoter activity and ExoS production (Fig 4E and 4F). These results implied that MiaB regulated T3SS gene expression not exclusively through LadS, additional pathways were recruited by MiaB to regulate T3SS gene expression besides the LadS.

### MiaB independently controlled *gacA*, *rsmY*, *rsmZ* to regulate T3SS gene expression

**Fig 5:**
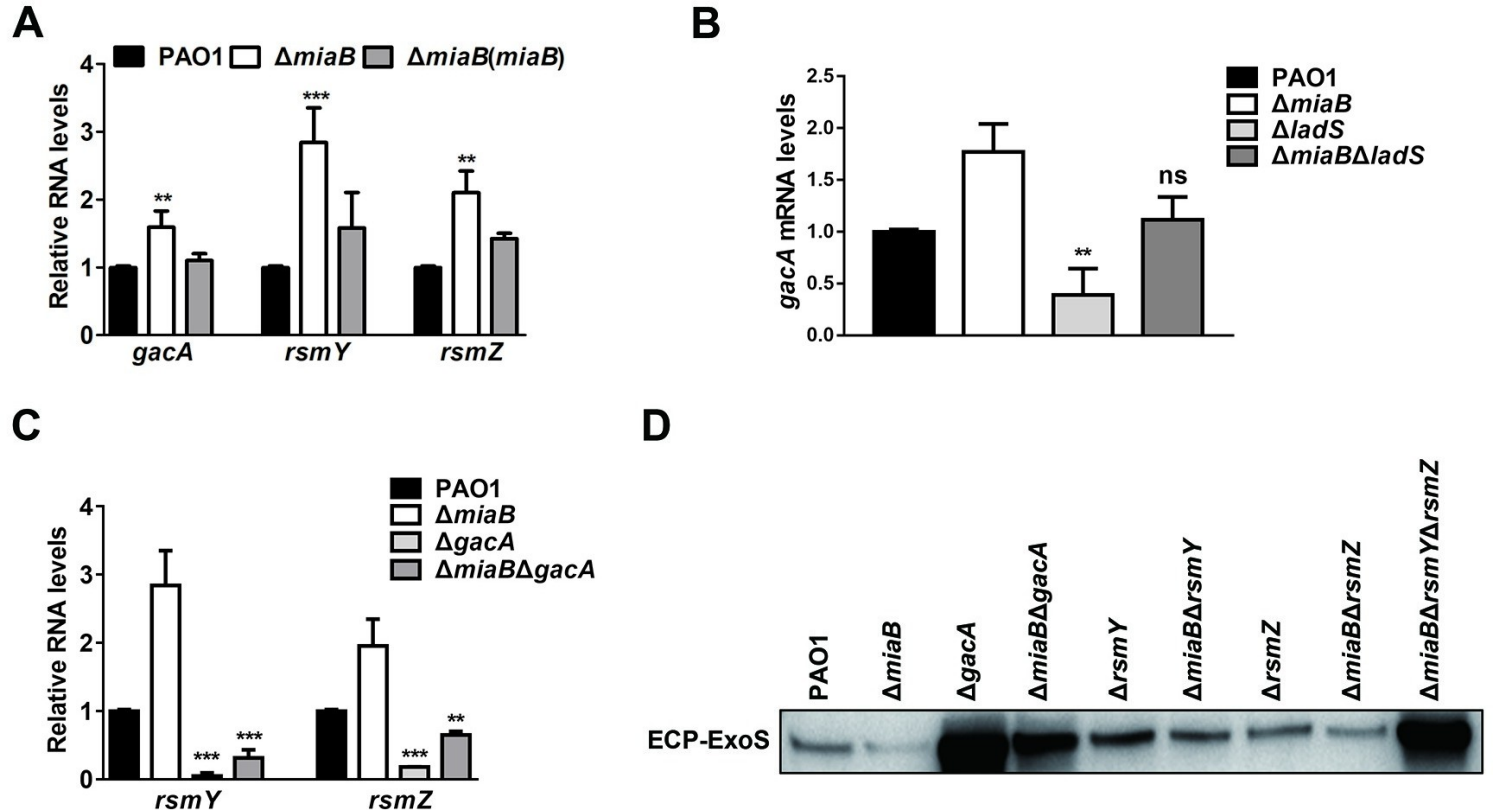
*gacA*, *rsmY*, *rsmZ* were repressed by MiaB to activate T3SS gene expression. (**A**) Relative expression of *gacA*, *rsmY* and *rsmZ* measured by RT-qPCR in the strains of wide-type PAO1, Δ*miaB*, and Δ*miaB* with *in trans* expression of *miaB*. (**B**) Relative expression of *gacA* measured by RT-qPCR in the strains of wide-type PAO1, Δ*miaB*, Δ*ladS* and Δ*miaB*Δ*ladS*. (**C**) Relative expression of *rsmY* and *rsmZ* measured by RT-qPCR in the strains of wide-type PAO1, Δ*miaB*, Δ*gacA* and Δ*miaB*Δ*gacA*. (**D**) Production of the ExoS protein measured by western blot in the wide-type PAO1 strain, Δ*miaB*, Δ*gacA*, Δ*rsmY*, Δ*rsmZ* and double or triple deletion mutants. ns, not significant; **, *P* < 0.01; ***, *P* < 0.001 compared to the wild-type PAO1 strain based on Student’s *t* test.

LadS is known to interact with GacS and positively regulate the two-component system GacS/GacA, which subsequently represses T3SS gene expression by inducing two small RNAs RsmY and RsmZ to sequester the T3SS activator RsmA [53]. Therefore, we hypothesized that MiaB might regulate T3SS gene expression directly through the components downstream of the LadS signaling pathway by circumventing LadS. To test this hypothesis, we examined the expression levels of *gacA*, *gacS*, *rsmA*, *rsmY* and *rsmZ* between the wild-type PAO1 strain and the Δ*miaB* mutant. RT-qPCR examination showed that expression of *gacA*, *rsmY*, *rsmZ* was significantly induced by the deletion of *miaB* and the induction could be abolished with *in trans* expression of *miaB* (Fig 5A), while deletion of *miaB* did not cause any difference on the expression levels of *gacS* and *rsmA* (S5 Fig). To further validate if MiaB could directly regulate *gacA*, *rsmY* and *rsmZ* independent of their upstream regulators, relative expression of *gacA* in the Δ*ladS* and Δ*miaB*Δ*ladS* strains was first measured. It showed that *gacA* was induced as well by deleting *miaB* in the Δ*ladS* background (Fig 5B). Similarly, expression of *rsmY* and *rsmZ* was induced as well when *miaB* was deleted in the Δ*gacA* background (Fig 5C). Together, these results indicated that MiaB independently regulated the components, i.e. *ladS*, *gacA*, *rsmY* and *rsmZ*, in the LadS-Gac/Rsm signaling pathway.

To evaluate the impact of *gacA*, *rsmY* and *rsmZ* on T3SS gene expression, we deleted them in the genetic background of the wild-type PAO1 strain and the mutant Δ*miaB*, respectively, and then determined the production of the T3SS effector ExoS by western blot assay in these strains. Same as the result of Δ*miaB*Δ*ladS* as shown in Fig 4F, Δ*miaB*Δ*gacA*, Δ*miaB*Δ*rsmY* and Δ*miaB*Δ*rsmZ* displayed an intermediate production level of ExoS between the single deletion mutants Δ*miaB* and Δ*gacA*, Δ*miaB* and Δ*rsmY*, Δ*miaB* and Δ*rsmZ*, respectively (Fig 5D). These results together validated that MiaB independently repressed the components of *ladS*, *gacA*, *rsmY* and *rsmZ*, which possibly released the T3SS activator RsmA and then led to the upregulation of T3SS gene expression.

### MiaB connected the Vfr and spermidine transporter-dependent regulatory systems to T3SS gene expression

**Fig 6:**
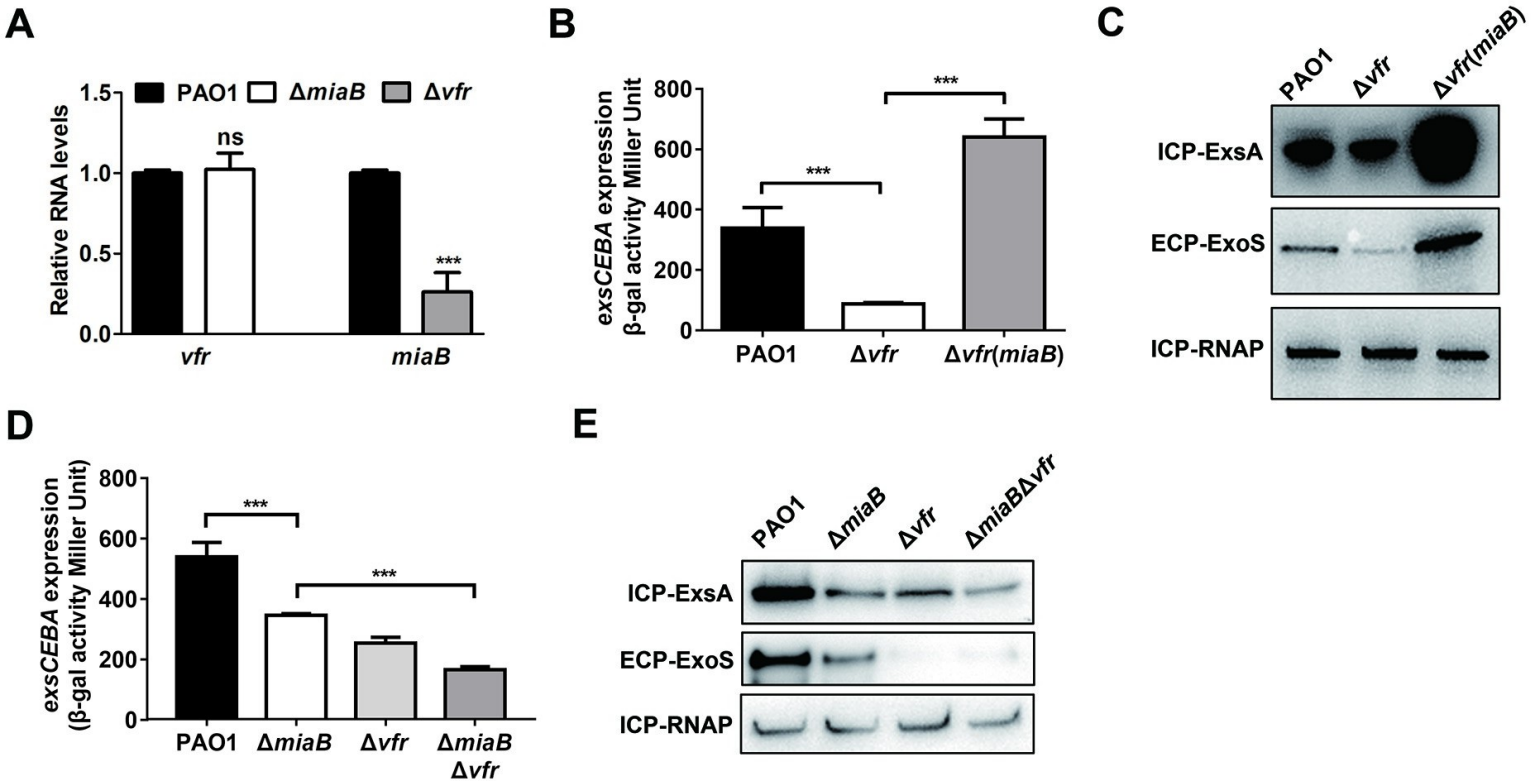
MiaB was positively regulated by the transcription regulator Vfr. (**A**) Expression of *vfr* and *miaB* in the strains of Δ*miaB* and Δ*vfr* relative to that in the wild-type PAO1 strain was measured by RT-qPCR, respectively. (**B**) *β*-galactosidase activity of the P*exsCEBA*-*lacZ* transcriptional fusion in the wide-type PAO1 strain, Δ*vfr*, and Δ*vfr* with *in trans* expression of *miaB*. (**C**) Productions of the intracellular ExsA and extracellular ExoS proteins measured by western blot in the wide-type PAO1 strain, Δ*vfr*, and Δ*vfr* with *in trans* expression of *miaB*. (**D**) *β*-galactosidase activity of the P*exsCEBA*-*lacZ* transcriptional fusion in the wide-type PAO1, Δ*miaB*, Δ*vfr*, and Δ*miaB*Δ*vfr* strains. (**E**) Productions of the intracellular ExsA and extracellular ExoS proteins measured by western blot in the wide-type PAO1, Δ*miaB*, Δ*vfr*, and Δ*miaB*Δ*vfr* strains. ns, not significant; ***, *P* < 0.001 compared to the wild-type PAO1 strain or the indicated group based on Student’s *t* test.

Vfr is a cAMP-dependent global regulator triggering *exsA* transcription in response to various stress conditions such as calcium-depletion [26]. To determine the potential regulatory relationship between MiaB and the Vfr/cAMP system on T3SS gene expression, we further constructed a Δ*vfr* mutant and determined the relative RNA levels of *vfr* and *miaB* in the mutant Δ*miaB* and Δ*vfr*, respectively, to the wild-type PAO1 strain. RT-qPCR analysis showed that the expression of *vfr* in Δ*miaB* was similar as that in the wild-type PAO1 strain, but expression of *miaB* in the mutant Δ*vfr* was decreased significantly compared to the wild-type PAO1 strain (Fig 6A), suggesting the expression of *miaB* was under the control of the Vfr/cAMP system. Quantitative *β*-galactosidase activity assay showed that the reduced transcription level of *exsCEBA* in the Δ*vfr* mutant could be restored by the *in trans* expression of *miaB* (Fig 6B). Consistently, reduced protein levels of the T3SS regulator ExsA and the effector ExoS were also recovered by the *in trans* expression of *miaB* (Fig 6C), implying that the reduced MiaB level in the Δ*vfr* mutant resulted in the decreased T3SS gene expression. Notably, deletion of *vfr* in the Δ*miaB* mutant led to the further decreased transcription level of *exsCEBA* and the translational levels of ExsA and ExoS compared to the Δ*miaB* mutant (Fig 6D and 6E), suggesting that Vfr/cAMP also modulated T3SS gene expression in a MiaB-independent manner which could be the direct control of the *exsA* transcription as previously reported [26]. These results together indicated that the positive regulation of the Vfr/cAMP system on T3SS gene expression was also connected by MiaB in addition to its direct stimulation on the *exsA* promoter.

**Fig 7:**
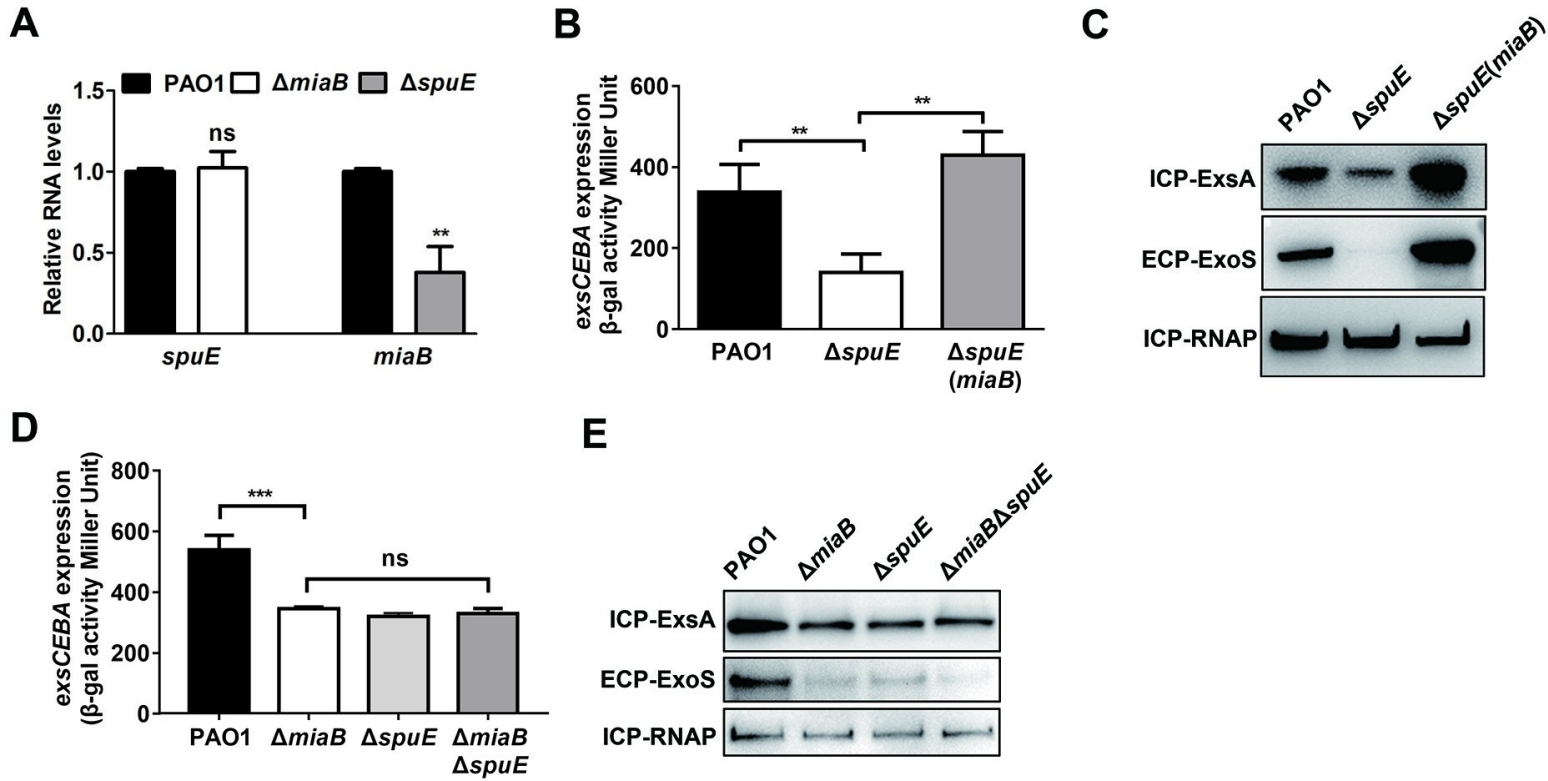
Disruption of the spermidine transporter by deleting *spuE* led to repressed expression of *miaB* and T3SS genes. (**A**) Expression of *spuE* and *miaB* in the strains of Δ*miaB* and Δ*vfr* relative to that in the wild-type PAO1 strain was measured by RT-qPCR, respectively. (**B**) *β*-galactosidase activity of the P*exsCEBA*-*lacZ* transcriptional fusion in the wide-type PAO1 strain, Δ*spuE*, and Δ*spuE* with *in trans* expression of *miaB*. (**C**) Productions of the intracellular ExsA and extracellular ExoS proteins measured by western blot in the wide-type PAO1 strain, Δ*spuE*, and Δ*spuE* with *in trans* expression of *miaB*. (**D**) *β*-galactosidase activity of the P*exsCEBA*-*lacZ* transcriptional fusion in the wide-type PAO1, Δ*miaB*, Δ*spuE*, and Δ*miaB*Δ*spuE* strains. (**E**) Productions of the intracellular ExsA and extracellular ExoS proteins measured by western blot in the wide-type PAO1, Δ*miaB*, Δ*spuE*, and Δ*miaB*Δ*spuE* strains. ns, not significant; **, *P* < 0.01; ***, *P* < 0.001 compared to the wild-type PAO1 strain or the indicated group based on Student’s *t* test.

Given that spermidine is an important host-derived signal and its transporter SpuDEFGH-dependent pathway plays an important role in T3SS activation in *P*. *aeruginosa* [42,54], we wondered if there was also a connection between MiaB and the spermidine transporter on T3SS gene expression in *P*. *aeruginosa*. To examine this, expression of a spermidine transporter gene *spuE* was first determined by RT-qPCR in the wild-type PAO1 strain and the mutant Δ*miaB*. Compared to the wild type, expression of *spuE* remained unchanged with the deletion of *miaB* (Fig 7A). On the contrary, the RNA level of *miaB* in Δ*spuE* was significantly lower than that in the wild-type PAO1 strain (Fig 7A), indicating the response of *miaB* expression to the spermidine transporter-dependent pathway. Same as the Vfr/cAMP system, *in trans* expression of *miaB* rescued the repressed *exsCEBA* promoter activity and the T3SS-associated proteins production in the Δ*spuE* mutant (Fig 7B and 7C). Moreover, further deletion of *spuE* in the Δ*miaB* mutant did not change the transcription level of *exsCEBA* and the productions of ExsA and ExoS (Fig 7D and 7E), implying that the regulation of the spermidine transporter-dependent signaling pathway on T3SS gene expression was exclusively connected by MiaB.

## Discussion

**Fig 8:**
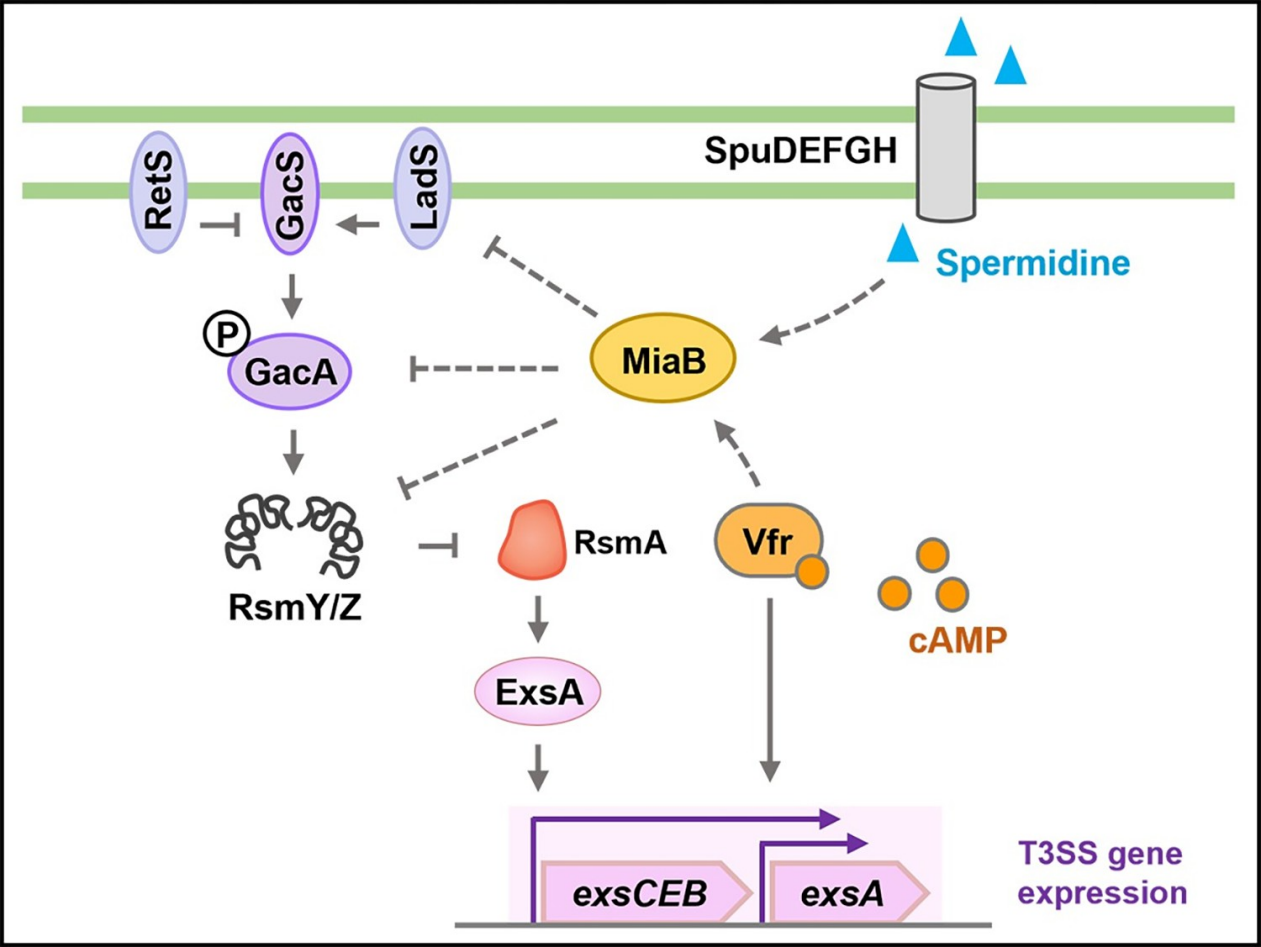
A diagram showing the MiaB-mediated regulation of T3SS in *P*. *aeruginosa*. The adenosine tRNA methylthiotransferase MiaB was upregulated by the cAMP-dependent regulator Vfr and the spermidine transporter-dependent pathway. MiaB independently repressed the expression of *ladS*, *gacA*, *rsmY* and *rsmZ* in the LadS-Gac/Rsm signaling pathway, which released RsmA to activate the transcription of *exsCEBA* and T3SS gene expression through the intrinsic regulator ExsA. Solid lines indicate direct regulations while dashed lines indicate probable indirect regulations. Arrows and T-shaped symbols indicate upregulation (activation) and repression, respectively.

Although great progress has been made to advance our understanding of the function and structure of T3SS in *P*. *aeruginosa*, regulatory networks that modulate T3SS gene expression especially the pathways transducing environmental signals to T3SS gene expression remain largely undetermined. T3SS in *P*. *aeruginosa* is usually expressed under the direct control of a master regulator ExsA from the well-characterized intrinsic ExsECDA partner-switching cascade while this regulatory module frequently cooperates with many extrinsic regulatory pathways in response to different signals such as spermidine, glutathione, Cis-2-dodecenoic acid (BDSF), direct host cell contact, serum and calcium depletion [42,55,56]. In the present study, we showed that the adenosine tRNA methylthiotransferase MiaB was upregulated by the cAMP-dependent regulator Vfr and the spermidine transporter-dependent pathway and its upregulation was essential for the activation of T3SS gene expression through the LadS-Gac/Rsm signaling pathway and the T3SS master regulator ExsA (Fig 8). These findings unveiled an important player MiaB, which connects environmental cues to modulate T3SS gene expression in *P*. *aeruginosa*.

Transposition-based screening facilitated us to discover a positive T3SS regulator PA3980 (MiaB) which was functionally characterized as an adenosine tRNA methylthiotransferase essential for the i^6^A modification. MiaB together with another tRNA prenyltransferase MiaA constitute a two-step tRNA modification process to catalyze one of the most common tRNA modification processes at position 37 (A37) [57]. The first step of A37 modification is conducted by MiaA, which catalyzes the addition of a prenyl group onto the *N*^6^-nitrogen of A37 to generate i^6^A. MiaB is responsible for the second step to synthesize the hypermodified nucleoside ms^2^i^6^A [49,58–60]. Levels of tRNA modification have been described as an adaptive strategy to change the proteome profiles in response to different environmental stimuli and stresses, and the dynamics of tRNA modification is known to contribute to the virulence of pathogens [61,62]. For example, the tRNA uridine 5-carboxymethylaminomethyl modification enzyme GidA post-transcriptionally regulates the Rhl quorum-sensing system and the tRNA-thiolating protein TtcA participates in the hydrogen peroxide-mediated stress protection and pathogenicity in *P*. *aeruginosa* [63,64]. tRNA modification generally occurs at the post-transcriptional stage to ensure the translational fidelity [62]. This study, for the first time to our knowledge, demonstrated that the adenosine tRNA methylthiotransferase MiaB is a novel regulator of T3SS gene expression in *P*. *aeruginosa* and, interestingly, MiaB-mediated regulation of T3SS gene expression is independent of tRNA modifications.

Regulation of T3SS by MiaB was interconnected with other well-characterized T3SS regulatory systems. Firstly, MiaB was shown to be an extrinsic T3SS regulator owing that its regulation was dependent on the intrinsic regulator ExsA. Loss of *exsA* completely abolished T3SS gene expression even with the *in trans* expression of MiaB. Further explorations elucidated that MiaB controlled T3SS gene expression through the LadS-Gac/Rsm signaling pathway. Expression of *ladS*, *gacA*, *rsmY* and *rsmZ* within the signaling pathway could be induced by deletion of MiaB while expression of another three components RsmA, GacS and RetS in the LadS/RetS/Gac/Rsm system were not influenced by MiaB. RsmA is the global post-transcriptional regulator directly activating T3SS gene expression and RetS inversely regulates Gac/Rsm systems with LadS [33, 36, 37], which are located at the most downstream and upstream of the Gac/Rsm system, respectively. How MiaB specifically modulated the transcription of *ladS*, *gacA*, *rsmY* and *rsmZ* but not *rsmA*, *gacS* and *retS* in the same signaling pathway is unclear. It was also interesting to note that MiaB could independently regulate *ladS*, *gacA*, *rsmY* and *rsmZ*. Because deletion of *miaB* in the Δ*gacA* background further increased expression of *rsmY* and *rsmZ* and decreased production of the T3SS protein ExoS, and similarly, deletion of *miaB* in the Δ*ladS* background still increased transcription of the downstream gene *gacA* and decreased production of ExoS.

Our results excluded the involvement of modified nucleosides in mediating the regulation of MiaB on T3SS gene expression, but how MiaB directly or indirectly controlled the expression of genes involved in modulating T3SS gene expression remains unclear. This could be achieved by the MiaB protein to physically interact with other transcription factors or function as a transcription factor to specifically recognize and bind to target promoter sequences. For example, we noticed the presence of direct *in vitro* interaction between MiaB and Vfr by using the pull-down assay (S6 Fig). Therefore, MiaB might serve as a partner protein that is essential to activate the T3SS regulator Vfr and possibly other transcription factors including those involved in regulating *ladS*, *gacA*, *rsmY* and *rsmZ*. In the future, transcriptome-wide examinations to explore the *miaB* regulon and high-throughput detections for the interactions between MiaB and its targeted promoter sequences or transcription factors may help to comprehensively elucidate its mechanisms on modulating T3SS gene expression.

In addition to the direct interaction between MiaB and Vfr, MiaB was found to be positively regulated by Vfr at transcriptional level. Its transcription was also regulated by the spermidine transporter-dependent signaling pathway. Spermidine is abundant in host cells [42, 54] and the activity of Vfr depends on cAMP whose level in *P*. *aeruginosa* is influenced by diverse stresses such as calcium limitation at the host-pathogen interface [65]. These findings thus establish that MiaB is capable of connecting these environmental cues to the activation of T3SS gene expression. Association of MiaB in modulation of T3SS has not yet been reported in other bacterial species, except a study showed that disruption of the T3SS positive regulator IscR caused about 2.0-fold upregulation of MiaB in a food-borne pathogen *Yersinia pseudotuberculosis* [66]. IscR is an Rrf2 family transcription regulator and has been studied extensively in *E*. *coli*. Its DNA-binding activity is dependent on coordination of an iron-sulfur [2Fe-2S] cluster, which is affected by various environmental cues, such as iron starvation, oxidative stress, and oxygen limitation [67–69]. However, whether MiaB is involved in the IscR-dependent regulation of T3SS in *Y*. *pseudotuberculosis* remains to be further investigated. Identification of MiaB as a key player in modulation of T3SS gene expression by integrating different environmental cues not only advanced our understanding on the complicated and sophisticated T3SS regulatory networks in *P*. *aeruginosa* but may also provide useful clues to elucidate its roles in other bacterial pathogens.

## Materials and methods

### Bacterial strains and growth conditions

Strains used in this study are listed in S1 Table. *P*. *aeruginosa* PAO1 and its derivatives were routinely grown at 37°C in normal Luria-Bertani (LB) medium or LB medium (LBN) supplemented with the calcium-chelating reagent nitrilotiracetic acid (NTA) at a final concentration of 7.5 mmol/L to induce T3SS. Minimal medium (MMN: 25 mM KH_2_O_4_, 95 mM NH_4_Cl, 50 mM monosodium glutamate, 110 mM disodium succinate, 10 mM trisodium nitriloacetate, 2.5% glycerol, 5 mM MgSO_4_, and 18 uM FeSO_4_) was also used as indicated [70]. The following antibiotics were supplemented to the culture medium when necessary: gentamicin at 50 μg/ml, kanamycin at 50 μg/ml, ampicillin at 100 μg/ml.

### Construction of mutants and plasmids for *in trans* expression

Plasmids constructed and used in this study are listed in S1 Table, and primers for plasmid construction are listed in S2 Table. For gene deletion, 500-bp upstream and 500-bp downstream region of target genes were amplified and ligated into the linearized pK18mobsacB vector at the restriction sites of BamHI, HindIII or EcoRI by One Step Cloning Kit (Vazyme Biotech, Nanjing, China). Triparental mating were performed with a helper strain HB101 (pRK2013) to deliver the constructed plasmids into the recipient strain. Recovered colonies were counter-selected by 5%~10% (wt/vol) sucrose in the plates. Mutants were confirmed by PCR and sequencing. For complementation assays, coding regions with the native promoters were amplified from PAO1 genomic DNA and cloned into the plasmid pBBR1-MCS5 which was pre-digested by the restriction enzymes BamHI, HindIII or EcoRI. The generated plasmids were introduced into mutants by triparental mating for *in trans* expression of the target genes.

### Promoter activity assays

For the *lacZ* reporter, overnight bacterial cultures were 1:100 diluted in fresh LBN or MMN and grown at 37°C until OD_600_ reaching 1.2–1.5. Cells were collected and *β*-galactosidase activity was measured as previously described [71]. Results were shown as the averages of miller units (MU) from at least three independent experiments. For the GFP reporter, promoter regions of *ladS* and *retS* were first amplified and cloned into the promoterless-*gfp* reporter pPROBE-NT to obtain pLadS-*gfp* and pRetS-*gfp*. After the constructed plasmids were introduced into wild-type PAO1 and Δ*miaB* strains, fluorescence was measured by CytoFLEX flow cytometer (Beckman Coulter, Brea, CA, USA) following the previously described method [72]. Results were shown as relative fluorescence when OD_600_ reaching 1.2–1.5, and as relative fluorescence/OD_600_ at the different time-course. All experiments were conducted with three independent biological repeats.

### Cytotoxicity assay

Bacterial cytotoxicity was determined in A549 cells by measuring the activity of released lactate dehydrogenase (LDH) in the culture supernatants post infection with *P*. *aeruginosa* PAO1 and its derivatives. Briefly, A549 cells with final 1.5 × 10^4^ cells/well were seeded in 96-well plate containing Dulbecco’s Modified Eagle Medium (DMEM) with 10% fetal bovine serum (FBS) at 37°C in 5% CO_2_. Supernatants were removed and washed twice with PBS buffer. Overnight bacterial cultures were 1:100 diluted with fresh LB and grown at 37°C until OD_600_ of 1.0. Bacteria were resuspended in DMEM with 1% FBS. Bacteria infected A549 cells at a multiplicity of infection (MOI) of 50 and cytotoxicity was then determined according to the manufacturer’s instructions. All experiments were conducted with three independent biological repeats.

### Western blot analysis

Overnight cultures were 1:100 diluted in LBN and grown until OD_600_ at 1.2–1.5. 10 ml cell culture were harvested at a same OD_600_ value for each experiment. After the cell cultures were harvested and centrifuged, the supernatants and cell pellets were processed for the detection of extracellular secreted proteins and intracellular proteins, respectively. The supernatants were purified with a 0.2-μm filter and precipitated with trichloroacetic acid (TCA) at a final concentration of 10% (vol/vol). After the precipitates were centrifuged with ultrafiltration tubes and washed with acetone, they were resuspended in the SDS-loading buffer. The bacterial pellets were resuspended in PBS buffer supplemented with protease inhibitor and the cells were broken by sonification and then centrifuged to collect protein solutions. Proteins were separated by SDS-PAGE and transferred to polyvinylidene difluoride (PVDF) membranes by electroblotting. Membranes were blocked by Blocking buffer (5% milk PBST) and hybridized with mouse polyclonal antibodies against ExsA, ExoS, PcrV (Our lab). Proteins were detected using the ECL kit (Abbkine) according to the manufacture’s protocol. Each experiment was repeated at least twice, and a representative result was selected and shown.

### Pull-down assay

The open reading frame of *miaB* and *vfr* were amplified from the PAO1 genome with primers listed in S2 Table and cloned into vectors pGEX-6p-1 and pET-32a, respectively. The constructs were transformed into *E*. *coli* BL21 and BL21(DE3) for expression of GST-tagged MiaB and His-tagged Vfr proteins. Pull-down assay was performed following the protocol provided by Glutathione Beads 4FF (Bio Basic Inc.). Briefly, Glutathione Beads 4FF was firstly mixed with the whole cell lysate of BL21 which expresses the GST-tagged MiaB protein and incubated at 4°C for 2 h. After unbound proteins were removed, whole cell lysate of BL21(DE3) which expresses the His-Vfr protein was added and incubated at room temperature (~25°C) for 1 h. After washing, proteins were eluted and loading buffer was then added. Samples were heated at 95°C for 5 min and supernatants were collected for SDS-PAGE and western blot analysis.

### Total RNA isolation, reverse transcription, and quantitative PCR

Overnight bacterial cultures were 1:100 diluted and grown to OD_600_ of 1.0. Total RNA was isolated using the RiboPure RNA purification kit, bacteria (Thermo Fisher Scientific) following the manufacturer’s protocol and the purity and quality of RNA were determined by agarose gel electrophoresis. RNA concentration was measured by NanoDrop 2000C (Thermo Fisher Scientific). 2 μg RNA was used for cDNA synthesis by TransScript First-Strand cDNA Synthesis kit (TransGen Biotech) following the protocol provided by the manufacturer. Quantitative PCR (qPCR) was performed by using PowerUp SYBR green master mix (Thermo Fisher Scientific) in a QuantStudio 6 Flex real-time PCR system (Applied Biosystems). The *rpoD* gene was selected as the internal reference for data analysis. The results were presented as the mean of three independent biological repeats.

### Nucleotide modification analysis

tRNA was isolated from total RNA samples by Urea-PAGE electrophoresis. tRNA with the size of 60–90 nt was purified by ethanol precipitation. Purified tRNA was quantified using Qubit RNA HS Assay kit (Thermo Fisher Scientific). tRNA was hydrolyzed to single nucleosides and then nucleosides were dephosphorylated. Pretreated nucleosides solution was deproteinized using the Satorius 10,000-Da MWCO spin filter. Analysis of nucleoside mixtures was performed on Agilent 6460 QQQ mass spectrometer with an Agilent 1260 HPLC system. All experiment were conducted with three independent biological repeats.

## Supporting information

S1 Table Strains and plasmids used in this study. (PDF)

S2 Table Primers used in this study. (PDF)

S1 Fig Sequence alignment between PA3980 in *P*. *aeruginosa* PAO1 and MiaB from *E*. *coli* (ECO) and *S*. typhimurium (STM). PA3980 from *P*. *aeruginosa* PAO1 shared 68.03% and 68.48% amino acid sequence identity with MiaB from *E*. *coli* and *S*. typhimurium, respectively.
(TIF)

S2 Fig MiaA and i^6^A were not involved in regulating *exsCEBA* transcription. (**A**) LC-MS measurement of the tRNA A37 *N*^6^-isopentenyladenosine (i^6^A) in the wide-type PAO1 strain and Δ*miaA* mutant. (**B**) *β*-galactosidase activity of the P*exsCEBA*-*lacZ* transcriptional fusion in the wide-type PAO1 strain, Δ*miaA*, and Δ*miaB* strains. ns, not significant, ***, *P* < 0.001 compared to the wild-type PAO1 strain based on Student’s *t* test.
(TIF)

S3 Fig *β*-galactosidase activity of the P*exsA*-*lacZ* transcriptional fusion in the wide-type PAO1 strain, Δ*miaB*, and Δ*miaB* with *in trans* expression of *miaB*. ns, not significant compared to the wild-type PAO1 strain based on Student’s *t* test.
(TIF)

S4 Fig Growth curves of the wild-type PAO1 strain and the Δ*miaB* mutant. (TIF)

S5 Fig Relative expression of *gacS* and *rsmA* in the wild-type PAO1 and Δ*miaB* mutant measured by RT-qPCR. ns, not significant compared to the wild-type PAO1 strain based on Student’s *t* test.
(TIF)

S6 Fig Pull-down assay indicated the presence of interaction between the MiaB and Vfr proteins. Pull-down assay was performed using glutathione beads with whole cell lysate of *E*. *coli* BL21 which expresses the GST-tagged MiaB protein (GST-MiaB) or/and whole cell lysate of BL21(DE3) which expresses the His-tagged Vfr protein (His-Vfr). GST-tagged MiaB was analyzed by immunoblotting with anti-GST antibody (upper panel) while the His-tagged Vfr protein was analyzed by immunoblotting with anti-His antibody (lower panel).
(TIF)
