# Supplementary material for: tRNA modification enzyme MiaB connects environmental cues to activation of Pseudomonas aeruginosa type III secretion system
Source: PLoS Pathog. 2022 Dec 5;18(12):e1011027. doi: 10.1371/journal.ppat.1011027 (PMC9754610; doi:10.1371/journal.ppat.1011027)
Supplement: S1 Table — (PDF) [file ppat.1011027.s001.pdf]

S1 Table. Strains and plasmids used in this study

Strains or plasmids	Description	Source
**Strains**		
E. coli DH5α	F-φ80d *lacZ*ΔM15 Δ(*lacZ*YA-*arg*F) U169 end A1 recA1 *hsd*R17($r_k^- m_k^+$) *sup*E44λ- thi-1 *gyr*A96 *rel*A1 *pho*A for plasmid transformation	Lab collection
BL21	*E. coli* B F^- dcm ompT hsdS ($rB^- mB^-$) gal $[malB^+]_{k\text{-}12}\lambda^s$	Lab collection
BL21(DE3)	F^- *omp*T *hsd*S_B ($r_B^- m_B^-$) *gal dcm* (DE3)	Lab collection
pRK2013	*Thr leu thi recA hsdR hsdM pro*, Km^r	Lab collection
P. aeruginosa PAO1	Prototrophic laboratory strain	Lab collection
PAO1pClacZ	*lacZ* fused to the *exsCEBA* promoter and integrated at the *attB* site of the PAO1 chromosome	Lab collection
PAO1pAlacZ	*lacZ* fused to the *exsA* promoter and integrated at the *attB* site of the PAO1 chromosome	This study
Δ*miaB*	*PA3980* or *miaB* in-frame deletion mutant from PAO1	This study
Δ*miaA*	*miaA* in-frame deletion mutant from PAO1	This study
Δ*exsA*	*exsA* in-frame deletion mutant from PAO1	This study
Δ*gacA*	*gacA* in-frame deletion mutant from PAO1	This study
Δ*ladS*	*ladS* in-frame deletion mutant from PAO1	This study
Δ*rsmY*	*rsmY* in-frame deletion mutant from PAO1	This study
Δ*rsmZ*	*rsmZ* in-frame deletion mutant from PAO1	This study
Δ*miaB*Δ*gacA*	*miaB* and *gacA* in-frame deletion mutant from PAO1	This study
Δ*miaB*Δ*ladS*	*miaB* and *ladS* in-frame deletion mutant from PAO1	This study
Δ*miaB*Δ*rsmY*	*miaB* and *rsmY* in-frame deletion mutant from PAO1	This study
Δ*miaB*Δ*rsmZ*	*miaB* and *rsmY* in-frame deletion mutant from PAO1	This study
Δ*miaB*Δ*rsmY*Δ*rsmZ*	*miaB*, *rsmY*, *rsmZ* in-frame deletion mutant from PAO1	This study
Δ*vfr*	*vfr* in-frame deletion mutant from PAO1	This study
Δ*spuE*	*spuE* in-frame deletion mutant from PAO1	This study
Δ*miaB*Δ*vfr*	*vfr* in-frame deletion mutant from Δ*miaB*	This study

Δ*miaB*Δ*spuE*	*spuE* in-frame deletion mutant from Δ*miaB*	This study
Δ*miaB*pClacZ	*miaB* in-frame deletion mutant from PAO1pClacZ	This study
Δ*miaA*pClacZ	*miaA* in-frame deletion mutant from PAO1pClacZ	This study
Δ*exsA*pClacZ	*exsA* in-frame deletion mutant from PAO1pClacZ	This study
Δ*ladS*pClacZ	*ladS* in-frame deletion mutant from PAO1pClacZ	This study
Δ*miaB*Δ*ladS*pClacZ	*speA* and *potD* in-frame deletion mutant from PAO1pClacZ	This study
Δ*vfr*pClacZ	*vfr* in-frame deletion mutant from PAO1pClacZ	This study
Δ*spuE*pClacZ	*spuE* in-frame deletion mutant from PAO1pClacZ	This study
Δ*miaB*Δ*vfr*pClacZ	*vfr* in-frame deletion mutant from Δ*miaB*pClacZ	This study
Δ*miaB*Δ*spuE*pClacZ	*spuE* in-frame deletion mutant from Δ*miaB*pClacZ	This study
Δ*miaB*pAlacZ	*miaB* in-frame deletion mutant from PAO1pAlacZ	This study
**Plasmids**		
pBBR1-MCS5	Broad-host-range expression vector, Gm^r	Lab collection
pBBR1-*miaB*	*miaB* encoding region cloned in pBBR1-MCS5, Gm^r	This study
pBBR1-*vfr*	*vfr* encoding region cloned in pBBR1-MCS5, Gm^r	This study
pBBR1-*spuE*	*spuE* encoding region cloned in pBBR1-MCS5, Gm^r	This study
pBBR1-*ladS*	*ladS* encoding region cloned in pBBR1-MCS5, Gm^r	This study
pPROBE-NT	Promoterless *gfp* transcriptional reporter plasmid, Gm^r	This study
$pRetS_{gfp}$	*gfp* transcriptional fusion with upstream region of *retS*	This study
$pLadS_{gfp}$	*gfp* transcriptional fusion with upstream region of *ladS*	This study
pET-*vfr*	*vfr* fused to His in frame in pET-32a, Amp^r	This study
pGEX-*miaB*	*miaB* fused to GST gene in frame in pGEX-6p-1, Amp^r	This study
