# Supplementary material for: tRNA modification enzyme MiaB connects environmental cues to activation of Pseudomonas aeruginosa type III secretion system
Source: PLoS Pathog. 2022 Dec 5;18(12):e1011027. doi: 10.1371/journal.ppat.1011027 (PMC9754610; doi:10.1371/journal.ppat.1011027)
Supplement: S2 Table — (PDF) [file ppat.1011027.s002.pdf]

S2 Table. Primers used in this study

Primers	Sequence
**For in-frame gene deletion**	
miaB-up-F	gagctcggtacccggggatccACGCCCTTCGCGCCGCTG
miaB-up-R	ccGCGCTTCGTCGGACAGTTC
miaB-down-F	aactgtccgacgaagcgcGGCCGACGACCGTACGGC
miaB-down-R	acgacggccagtgccaagcttGCCTTGCGCGACTTCAGG
miaA-up-F	gagctcggtacccggggatccAAGGCTTGCGTGGGCAGC
miaA-up-R	aattaaggtcCGGAACGCTCTTGGACAAAG
miaA-down-F	gagcgttccgGACCTTAATTTGTCCGTCTGTTTCC
miaA-down-R	acgacggccagtgccaagcttAATGCCACCGTTTCCGCG
exsA-up-F	gagctcggtacccggggatccCCTTCGATCTGGAGGTCGACA
exsA-up-R	ccgggctttcaaaaaacgATTATAAGAACCCCAACACTTCCC
exsA-down-F	atCGTTTTTTGAAAGCCCGGT
exsA-down-R	acgacggccagtgccaagcttGCAGGCTTTCCCACCAGC
gacA-up-F	gagctcggtacccggggatccCATCAGGAAGCAATCCTGGATC
gacA-up-R	aaaacggcgctcatGCTGCACCTCGTCGCGCA
gacA-down-F	tgcagcATGAGCGCCGTTTTCGACG
gacA-down-R	acgacggccagtgccaagcttCAGGGCCGCGTACGGTTG
rsmY-up-F	gagctcggtacccggggatccGGTGGCCACGTAGTTCGGG
rsmY-up-R	cctcgggcaataaGGTTTGAAGATTACGCATCTCTGC
rsmY-down-F	tcaaaccTTATTGCCCGAGGAAAACCG

rsmY-down-R	acgacggccagtgccaagcttCTGCTCACCGGCAACCTG
rsmZ-up-F	gagctcggtacccggggatccCGAGCTGCTGCAGGATGACG
rsmZ-up-R	gcaggcaaaCAGGAGTGATATTAGCGATTCCC
rsmZ-down-F	tatcactcctgTTTGCCTGCCGTTTTACTCG
rsmZ-down-R	acgacggccagtgccaagcttGCCCGCGGCAAGCTCTCG
vfr-up-F	gagctcggtacccggggatccCGGCCTCGAGGAAGGCTT
vfr-up-R	tttttcatgggtgctgtGCCCGAGTCCCGAAAGAA
vfr-down-F	ggcACAGCACCCATGAAAAAGGCC
vfr-down-R	acgacggccagtgccaagcttCGAGGCGCGGGCGATCGG
spuE-up-F	gagctcggtacccggggatccGCTGTATACCTTCCCCGACCTG
spuE-up-R	gaatttgaacGTCCGACTCCTGTTCCGGG
spuE-down-F	ggagtcggacGTTCAAATTCCGCCCGGT
spuE-down-R	acgacggccagtgccaagcttTTGCGCTTGGCGTACTGG
**For *in trans* complementation**	
pBBR1-*miaB*-F	gtcgacggtatcgataagcttATGGCCAAGAAGCTTTTCATCG
pBBR1-*miaB*-R	cgctctagaactagtggatccTCAATGCAGGGTGCTGTCG
pBBR1-*exsA*-F	gtcgacggtatcgataagcttATGCAAGGAGCCAAATCTCTTG
pBBR1-*exsA*-R	cgctctagaactagtggatccTCAGTTATTTTTAGCCCGGCA
pBBR1-*ladS*-F	gtcgacggtatcgataagcttGGCTTCGATGTCGGCTTGC
pBBR1-*ladS*-R	cgctctagaactagtggatccTCAGGCGGACTTGGTGACG
**For RT-qPCR**	
rpoD-F	GCGAAGAAGGAAATGGTC

rpoD-R	CGTAGGTGGAGAACTTGTA
miaB-F	TACCGTGCTGGAATACAA
miaB-R	AGATGAAGGAGAAGGAGAAG
exsA-F	CTGCCCAGTTTCTACAAG
exsA-R	CACAAGCAATTCCTTCAAC
exsC-F	CCGTTTCGATCTGCATTTC
exsC-R	GAAGCATTCGAGGGTCAG
exsD-F	AGGAAGACGATAAGCAGTA
exsD-R	CGGACTCACGATACAAAC
pcrV-F	CACGCTATATGGCTATGC
pcrV-R	GCTGAAGGTATCCAGATTG
popN-F	GGACATCCTCCAGAGTTC
popN-R	CGAAACGGAAACGTAGTG
pscN-F	GAGTTGCTGCTGAAGATC
pscN-R	GTAATCGCTGGTTTCGTG
pscP-F	CAGTTGCAGATCGAGATC
pscP-R	CTGGTTGAAGGTGAGTTG
pcrR-F	CTGATTCCCTGGTTTCTC
pcrR-R	CAACATCACGATCCAGAC
pscD-F	GTGCCCTATGTAGTGCTC
pscD-R	CCCTTTGAGATTGATGATGAAA
ladS-F	TGGTGATGCTGATCTACAA

ladS-R	AACGAAGCGATATAGAGGAT
gacA-F	ATCAAGGTCGTGGTAGTC
gacA-R	ATCTGCGGGCTGATATAG
gacS-F	GACTACCTCACGACCATC
gacS-R	CAGTTCCAGTTGCTTCTC
rsmY-F	GCCAAAGACAATACGGAAA
rsmY-R	GCAGACCTCTATCCTGAC
rsmZ-F	AGGGAACACGCAACC
rsmZ-R	CCGCCCACTCTTCAG
spuE-F	CAACATTCCATTGGCAAGA
spuE-R	TGTAATCGGTCCAGTTATAGAT
vfr-F	TATTACCCACACACCCAAA
vfr-R	GTTGAGGTAGCCGATGAT
**For pPROBR-NT fusion**	
NT-*retS*-F	caggtcgactctagaggatccCGATCATGGTCCGCCTGG
NT-*retS*-R	ctttagttagttagggaattcGGCGATCCGAAGCCGTAC
NT-*ladS*-F	caggtcgactctagaggatccGCTTCGATGTCGGCTTGCA
NT-*ladS*-R	ctttagttagttagggaattcAATCAGCCAGTGCCGCATG
**For protein expression**	
GST-*miaB*-F	ttccaggggcccctgggatccATGGCCAAGAAGCTTTTCATCG
GST-*miaB*-R	gtcacgatgcggccgctcgagTCAATGCAGGGTGCTGTCG
pET-*vfr*-F	atcggatccgaattcgagctcATGGTAGCTATTACCCACACACCC

pET-*vfr*-R	gtggtggtggtggtgctcgagTCAGCGGGTGCCGAAGAC
