# Supplementary figures and images for: tRNA modification enzyme MiaB connects environmental cues to activation of Pseudomonas aeruginosa type III secretion system
Source: PLoS Pathog. 2022 Dec 5;18(12):e1011027. doi: 10.1371/journal.ppat.1011027 (PMC9754610; doi:10.1371/journal.ppat.1011027)

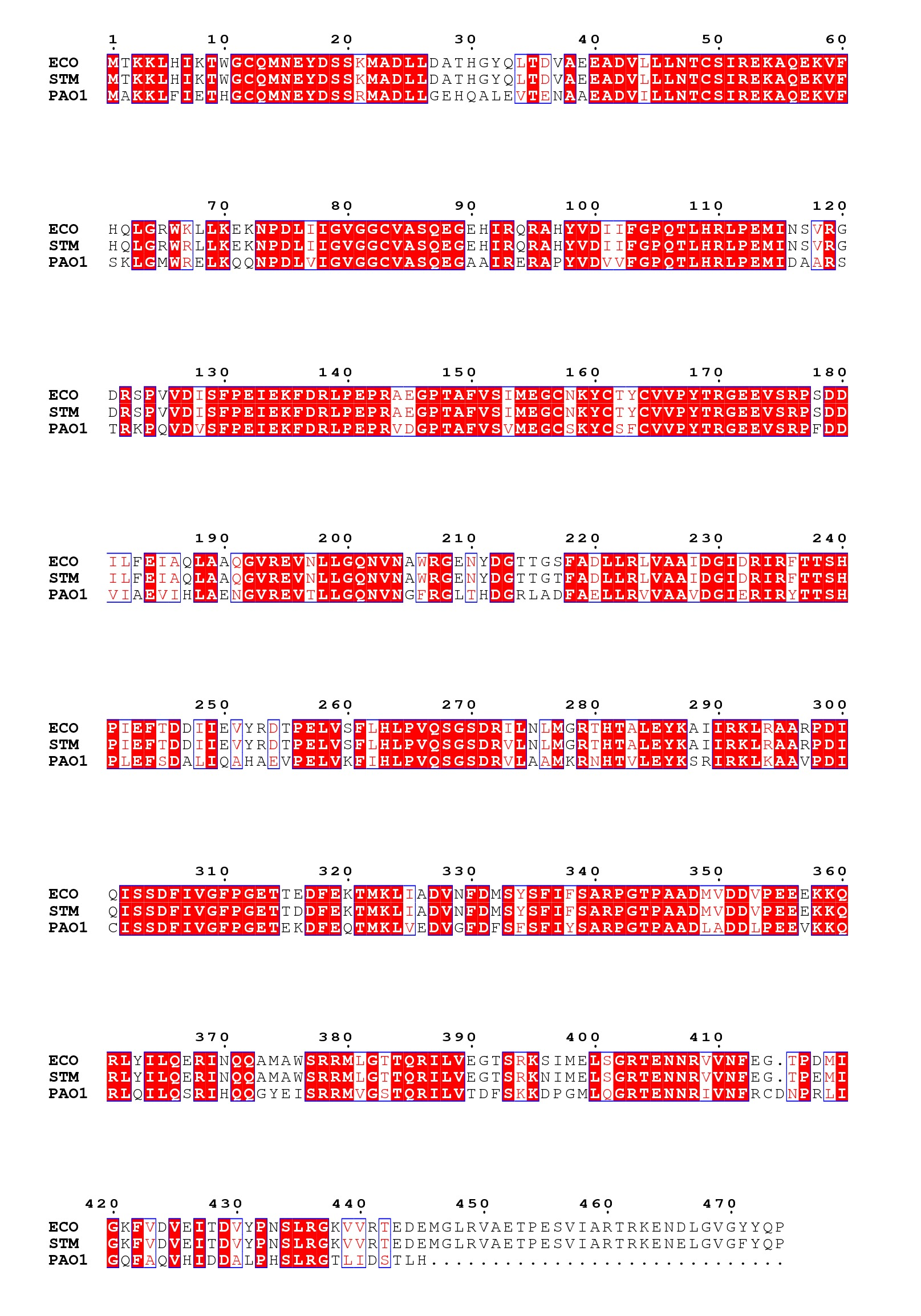

Supplement: S1 Fig — PA3980 from P. aeruginosa PAO1 shared 68.03% and 68.48% amino acid sequence identity with MiaB from E. coli and S. typhimurium, respectively. (TIF) [file ppat.1011027.s003.tif]

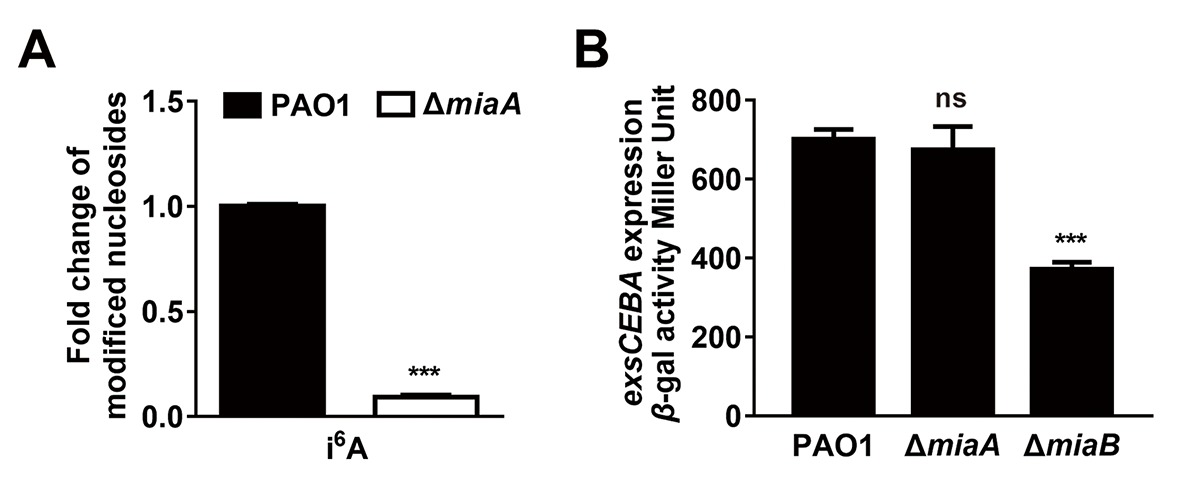

Supplement: S2 Fig — (A) LC-MS measurement of the tRNA A37 N6-isopentenyladenosine (i6A) in the wide-type PAO1 strain and ΔmiaA mutant. (B) β-galactosidase activity of the PexsCEBA-lacZ transcriptional fusion in the wide-type PAO1 strain, ΔmiaA, and ΔmiaB strains. ns, not significant, ***, P < 0.001 compared to the wild-type PAO1 strain based on Student’s t test. (TIF) [file ppat.1011027.s004.tif]

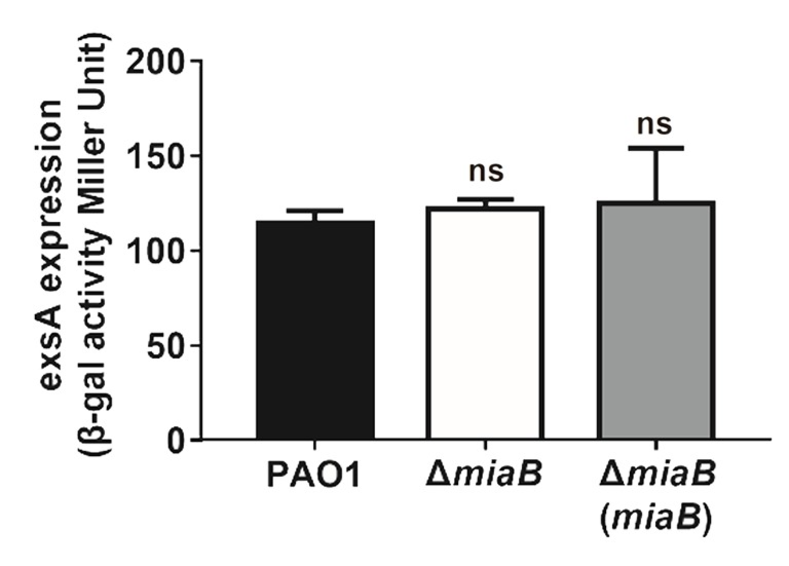

Supplement: S3 Fig — ns, not significant compared to the wild-type PAO1 strain based on Student’s t test. (TIF) [file ppat.1011027.s005.tif]

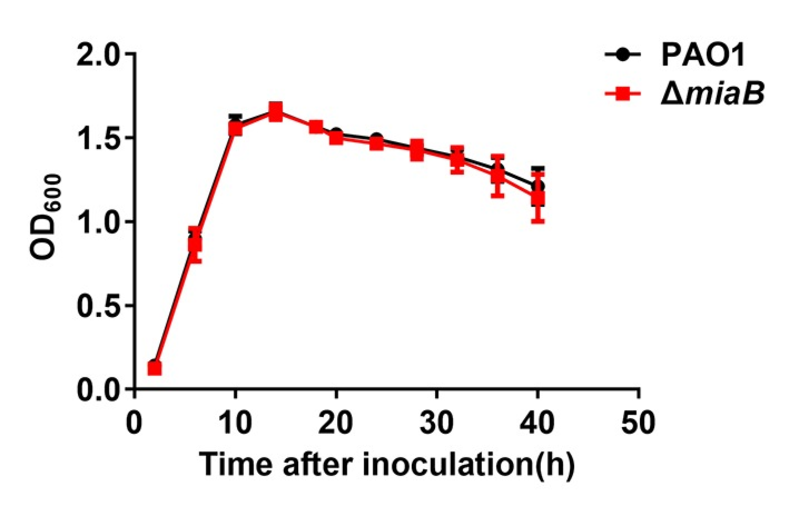

Supplement: S4 Fig — (TIF) [file ppat.1011027.s006.tif]

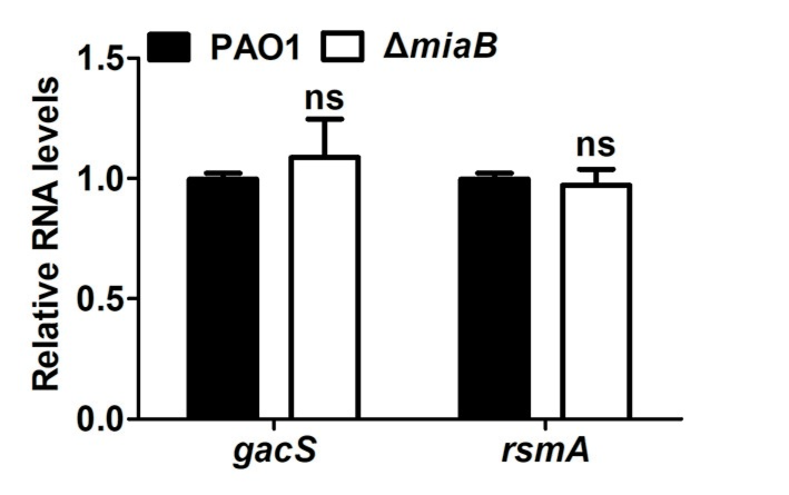

Supplement: S5 Fig — ns, not significant compared to the wild-type PAO1 strain based on Student’s t test. (TIF) [file ppat.1011027.s007.tif]

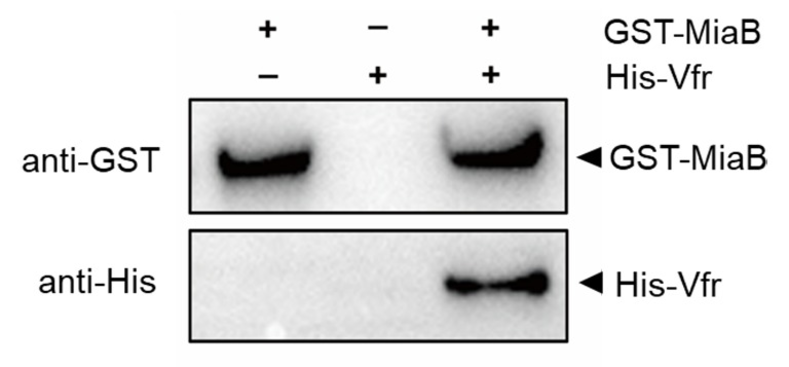

Supplement: S6 Fig — Pull-down assay was performed using glutathione beads with whole cell lysate of E. coli BL21 which expresses the GST-tagged MiaB protein (GST-MiaB) or/and whole cell lysate of BL21(DE3) which expresses the His-tagged Vfr protein (His-Vfr). GST-tagged MiaB was analyzed by immunoblotting with anti-GST antibody (upper panel) while the His-tagged Vfr protein was analyzed by immunoblotting with anti-His antibody (lower panel). (TIF) [file ppat.1011027.s008.tif]
